# A pathogenic tau fragment compromises microtubules, disrupts insulin signaling and induces the unfolded protein response

**DOI:** 10.1186/s40478-018-0651-9

**Published:** 2019-01-03

**Authors:** Tong Guo, Dina Dakkak, Teresa Rodriguez-Martin, Wendy Noble, Diane P. Hanger

**Affiliations:** King’s College London, Institute of Psychiatry, Psychology & Neuroscience, Department of Basic and Clinical Neuroscience, Maurice Wohl Clinical Neuroscience Institute (K1.24), 5 Cutcombe Road, London, SE5 9RX UK

**Keywords:** Tau, Tauopathy, Microtubule binding, Akt, Glycogen synthase kinase-3, Insulin, Unfolded protein response

## Abstract

Human tauopathies including Alzheimer’s disease, progressive supranuclear palsy and related disorders, are characterized by deposition of pathological forms of tau, synaptic dysfunction and neuronal loss. We have previously identified a pathogenic C-terminal tau fragment (Tau35) that is associated with human tauopathy. However, it is not known how tau fragmentation affects critical molecular processes in cells and contributes to impaired physiological function. Chinese hamster ovary (CHO) cells and new CHO cell lines stably expressing Tau35 or full-length human tau were used to compare the effects of disease-associated tau cleavage on tau function and signaling pathways. Western blots, microtubule-binding assays and immunofluorescence labeling were used to examine the effects of Tau35 on tau function and on signaling pathways in CHO cells. We show that Tau35 undergoes aberrant phosphorylation when expressed in cells. Although Tau35 contain the entire microtubule-binding region, the lack of the amino terminal half of tau results in a marked reduction in microtubule binding and defective microtubule organization in cells. Notably, Tau35 attenuates insulin-mediated activation of Akt and a selective inhibitory phosphorylation of glycogen synthase kinase-3. Moreover, Tau35 activates ribosomal protein S6 kinase beta-1 signaling and the unfolded protein response, leading to insulin resistance in cells. Tau35 has deleterious effects on signaling pathways that mediate pathological changes and insulin resistance, suggesting a mechanism through which N-terminal cleavage of tau leads to the development and progression of tau pathology in human tauopathy. Our findings highlight the importance of the N-terminal region of tau for its normal physiological function. Furthermore, we show that pathogenic tau cleavage induces tau phosphorylation, resulting in impaired microtubule binding, disruption of insulin signaling and activation of the unfolded protein response. Since insulin resistance is a feature of several tauopathies, this work suggests new potential targets for therapeutic intervention.

**Electronic supplementary material:**

The online version of this article (10.1186/s40478-018-0651-9) contains supplementary material, which is available to authorized users.

## Background

Tauopathies are a heterogeneous group comprising dementias and movement disorders, neuropathologically characterized by prominent intracellular accumulations of neurofibrillary tangles formed of tau in neurons and glia. Accumulating evidence suggests that the conversion of physiological tau to pathological tau plays a central role in the development of tauopathy. In particular, abnormal phosphorylation and fragmentation of tau have been proposed as important post-translational modifications that lead to pathogenic forms of tau [19]. In addition, a range of inter-related cellular processes, including microtubule disorganization [18, 55, 56], activation of the unfolded protein response (UPR) [22, 34, 52], activation of the nutrient sensor mammalian target of rapamycin complex 1 (mTORC1) [9, 49], and deficiencies in insulin signaling [41, 45], also promote cell dysfunction in tau-mediated neurodegeneration. However, the cellular events linking pathological changes in tau to cell dysfunction and the pathogenesis of tauopathies are largely unknown.

We previously described a 35 kDa C-terminal tau fragment (Tau35), lacking the N-terminus of tau but containing all four microtubule-binding repeats (4R), that is present in 4R tauopathies [53]. When expressed in transgenic mice, Tau35 induces several key features of tauopathy, including accumulation of abnormally phosphorylated tau, dysregulation of glycogen synthase kinase-3β (GSK3β) activity, progressive cognitive and motor deficits, and loss of synaptic proteins [7]. Here we have used a cell model to investigate the molecular mechanisms that are affected by Tau35 expression. Our findings suggest that aberrant tau cleavage may have a key role in disrupting physiological signaling pathways involved in the development of tauopathy.

## Materials and methods

### Plasmids

Plasmids encoding full-length 2N4R human tau (FL-tau) or Tau35 were generated in pcDNA 3.1D/V5-His-TOPO vector (Invitrogen), which carries a neomycin resistance gene, a V5 epitope, a 6 × His tag and the promoter from cytomegalovirus. The original plasmid encoding FL-tau in bacterial expression vector pRK172 was a kind gift from Professor Michel Goedert (MRC Laboratory of Molecular Biology, Cambridge, UK). cDNA sequences corresponding to FL-tau and Tau35 were each inserted into the multiple cloning site of the pcDNA 3.1D/V5-His-TOPO vector at BamHI-XbaI [12]. Alpha-tubulin N-acetyltransferase 1 (αTAT1) plasmid [2] was a kind gift from Professor Jacek Gaertig (University of Georgia, USA).

### Cell maintenance and transfection

Mycoplasma negative Chinese hamster ovary (CHO) cells, acquired from the European Collection of Authenticated Cell Cultures, were grown at 37 °C with 5% CO_2_ in Ham’s F12 medium supplemented with 10% fetal bovine serum, 2 mM L-glutamine, 100 units/mL penicillin and 100 μg/mL streptomycin (Thermo Fisher Scientific). 24 h before transfection, cells were plated at a density of 3.7 × 10^4^ cells/cm^2^ in 6-well or 12-well plates. CHO cells were transiently transfected with plasmids (2 μg plasmid/well for 6-well plate or 1 μg plasmid/well for 12-well plate) using jetPEI™ (Polyplus Transfection) according to the manufacturer’s instructions. 24 or 48 h after transfection, cells were processed for biochemical assays, or fixed for immunocytochemistry.

### Generation of stable CHO cell lines

CHO cells were transiently transfected with plasmids encoding either FL-tau or Tau35, as described above. Non-transfected CHO cells were included as controls. 48 h after transfection the medium was replaced by Ham’s F-12 medium as above, with the addition of 800 μg/mL G418 (Santa Cruz). After selection, G418-resistant cells were transferred to 145 mm diameter dishes for clonal isolation. Cell clusters were isolated using cloning cylinders (Sigma) and transferred to 6-well plates for clonal expansion. Further characterization of the G418-resistant cells was undertaken using western blots to examine the stable expression of tau protein and immunocytochemistry to assess the homogeneity of the cell lines. Clonal cells homogenously expressing 2N4R tau or Tau35, termed CHO-FL and CHO-Tau35, respectively, were selected and maintained in CHO cell growth medium without G418 at 37 °C in 5% CO_2_.

### Cell treatments

24 h before treatment, CHO-FL, CHO-Tau35 and untransfected CHO cells were seeded at a density of 3.7 × 10^4^ cells/cm^2^. For insulin treatment, cells were treated with 100 nM insulin (Sigma) for 30 min at 37 °C before washing in phosphate-buffered saline (PBS, 137 mM NaCl, 2.7 mM KCl, 8 mM Na_2_HPO_4_, 2 mM KH_2_PO_4_, pH 7.4). For LiCl treatment, cells were treated with 5 mM LiCl, or 5 mM NaCl (control) at 37 °C for 24 h, then washed with PBS. For thapsigargin treatment, cells were treated with 800 nM thapsigargin for 5 h at 37 °C, then washed with PBS. After treatment, cells were either scraped into ice-cold Tris-HCl buffer (50 mM Tris-HCl, pH 7.4, 150 mM NaCl, 1 mM Na_3_VO_4_, Complete protease inhibitor and Complete protease inhibitor cocktail [Roche]), lysed in 2× Laemmli sample buffer and heated at 95 °C for 10 min for analysis on western blots, or fixed for immunocytochemistry, as described below.

### In situ microtubule binding assay

In situ microtubule binding was assayed as described previously [40]. Briefly, 24 h before the experiment, CHO-FL and CHO-Tau35 cells were plated (3.7 × 10^4^ cells/cm^2^). Cells were rinsed with warm PBS and scraped into warm PIPES buffer (80 mM piperazine-N,N′bis-2-ethanesulfonic acid, pH 6.8, 1 mM guanosine-5′-triphosphate, 1 mM MgCl_2_, 1 mM ethylene glycol-bis(2-aminoethyl)-N,N,N′,N′-tetraacetic acid, 0.5% (*w*/*v*) Triton X-100 and 30% (*v*/v) glycerol) containing Complete protease inhibitor (Roche), 20 mM NaF, 0.5 μM okadaic acid (Merck), and 10 μM taxol (Sigma). Cell lysates were centrifuged at 5000 g for 10 min at ambient temperature, and an aliquot of the supernatant was retained (total, T). The remaining post-nuclear lysate was centrifuged at 100,000 g for 1 h at 37 °C. The supernatant (unbound fraction, U) was collected, and the pellet (bound fraction, B) was rinsed twice in PIPES buffer, pelleted at 100,000 g, and then resuspended in PIPES buffer. All fractions were suspended in 2× Laemmli sample buffer and heated at 95 °C for 10 min prior to analysis on western blots.

### Western blots

Proteins in cell lysates and sub-cellular fractions were separated on sodium dodecyl sulfate polyacrylamide gel electrophoresis. Electrophoresed proteins were transferred onto 0.45 μm nitrocellulose membranes. Membranes were blocked in Odyssey blocking buffer (Li-Cor Biosciences), 3% (*w*/*v*) dried skimmed milk in Tris-buffered saline/0.2% (*v*/*v*) Tween 20 (TBST), or 5% (*w/v*) bovine serum albumin in TBST for 30 min at ambient temperature, then incubated overnight at 4 °C in primary antibodies (Additional file 1: Table S1). After washing, membranes were incubated for 60 min at ambient temperature with the appropriate fluorophore-conjugated secondary antibody (Alexa Fluor® 680 goat anti-mouse immunoglobulin G (IgG) or IRDye™ 800 goat anti-rabbit IgG, Invitrogen). Antigens were visualized using an Odyssey® infrared imaging system (Li-Cor Biosciences). Images were analyzed using Li-Cor Image Studio Lite software (Li-Cor Biosciences).

### Immunocytochemistry

Cells on coverslips were washed 3 times in PBS, fixed for 10 min in 4% (*w*/*v*) paraformaldehyde at 37 °C or in ice-cold methanol at − 20 °C for 10 min. Paraformaldehyde-fixed cells were permeabilized using 0.25% (*v*/*v*) Triton X-100 for 10 min at ambient temperature and washed in PBS. Following incubation in blocking buffer (10% (*v/v*) fetal bovine serum in PBS, pH 7.4) for 30 min at ambient temperature. Cells were incubated in primary antibody overnight at 4 °C, followed by secondary antibody for 60 min at ambient temperature (Additional file 1: Table S1). Nuclei were stained using Hoechst 33342 (5 μg/mL bisbenzimide in PBS). Fluorescence microscopy was performed using a Leica DM5000B fluorescence microscope equipped with a 63×/1.25 immersion objective, and a digital camera (DFC360 FX, Leica) using Leica Application Suite Advanced Fluorescence Software (Leica). Images were processed and analyzed using ImageJ software [44].

### Quantification and statistical analysis

Quantitative analyses were performed using Microsoft Excel and GraphPad Prism 7. Normality for individual variables was determined by the Shapiro-Wilk test and analyzed using Student’s unpaired *t*-test or one-way analysis of variance (ANOVA) followed by Tukey’s post-hoc tests. Differences were considered statistically significant when *P* < 0.05.

## Results

### Abnormal phosphorylation of Tau35

**Fig. 1:**
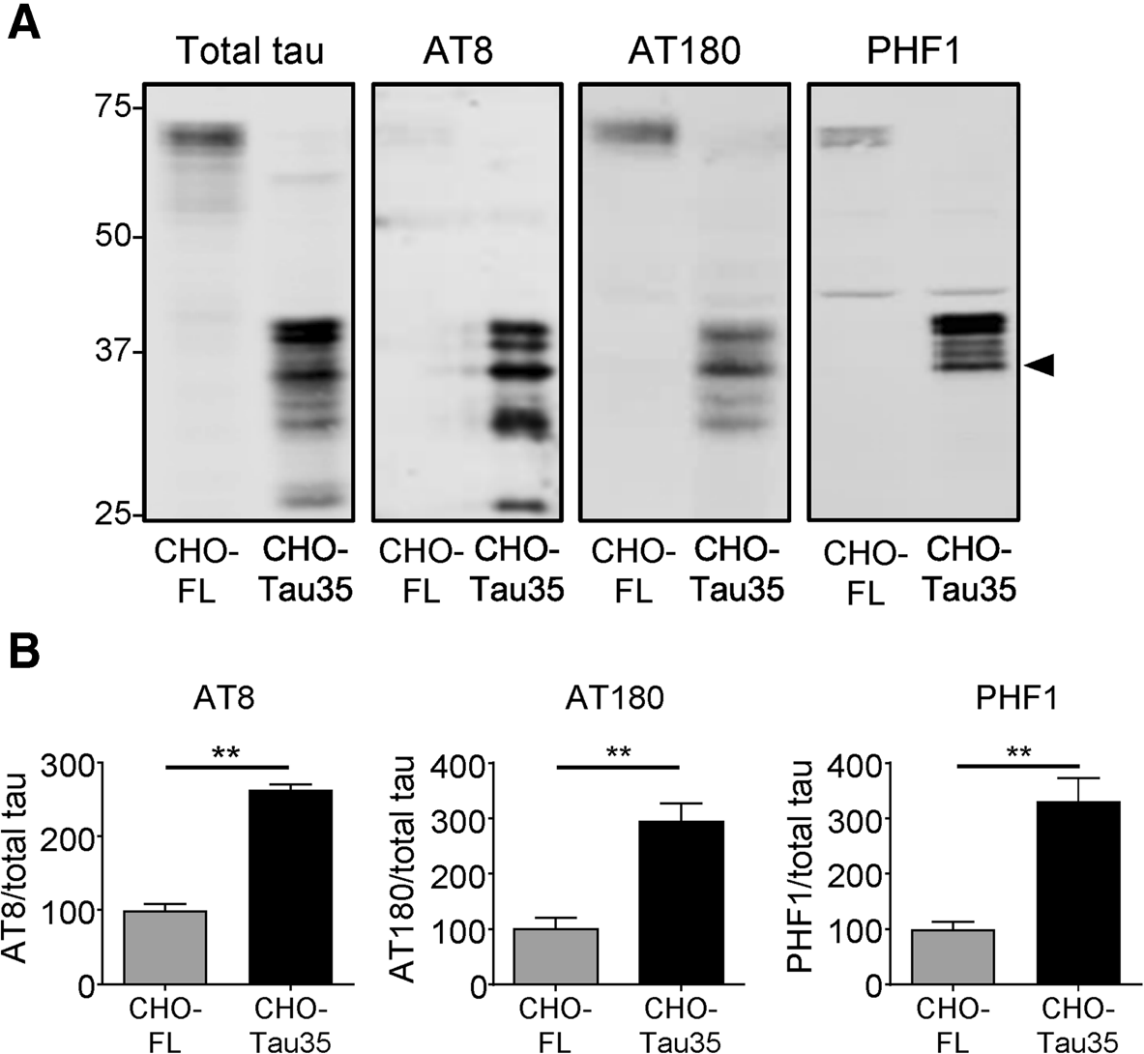
Abnormal phosphorylation of Tau35. **a** Western blots of CHO-FL and CHO-Tau35 cell lysates probed with antibodies against total and phosphorylated tau (AT8: pSer202/pThr205; AT180: pThr231; and PHF1: pSer396/pSer404). Molecular weight markers (kDa) are shown on the left. **b** Graphs show the ratio of phosphorylated/total tau in CHO-FL and CHO-Tau35 cells, standardized to CHO-FL cell values (100%). Values represent mean ± S.E.M., *n* = 3–4. Student’s t-test, **P < 0.01

Tau phosphorylation plays an important role in regulating tau function and aberrant tau phosphorylation is widely acknowledged as a key component of tauopathy pathogenesis [20, 32]. Therefore, we examined the effect of N-terminal cleavage on the phosphorylation status of tau in cells. Lysates of CHO cells stably expressing either full-length (2N4R isoform) human tau (CHO-FL) or Tau35 (CHO-Tau35) were analyzed on western blots using antibodies to phosphorylated tau (AT8, pSer202/pThr205; AT180, pThr231; and PHF1, pSer396/pSer404) and total tau (Fig. 1a). Antibodies recognizing phosphorylated tau exhibited only modest (AT180, PHF1) or negligible (AT8) immunoreactivity with FL-tau, whereas all tau antibodies recognized multiple Tau35 bands (Fig. 1a). The total tau antibody labelled predominant species of 39 (doublet) and 35 kDa (Fig. 1a, arrowhead), together with minor tau bands at 30 and 26 kDa, indicating the presence of modified and degraded tau in CHO-Tau35 cells. All three phospho-dependent tau antibodies detected the 39 and 35 kDa Tau35 species, although the 39 kDa doublet appeared as a single band with AT180, suggesting that tau species derived from CHO-Tau35 cells undergo phosphorylation at multiple residues. Tau species smaller than 35 kDa were not labeled by PHF1, indicating that Tau35 may be subject to C-terminal cleavage in cells. Quantification of the blots showed that compared to FL-tau, Tau35 phosphorylation increased approximately 2–3-fold at each of the epitopes examined (Fig. 1b). These results demonstrate that N-terminally cleaved tau is consistently and abnormally phosphorylated at several disease-relevant epitopes.

### Disrupted microtubule organization in CHO-Tau35 cells

**Fig. 2:**
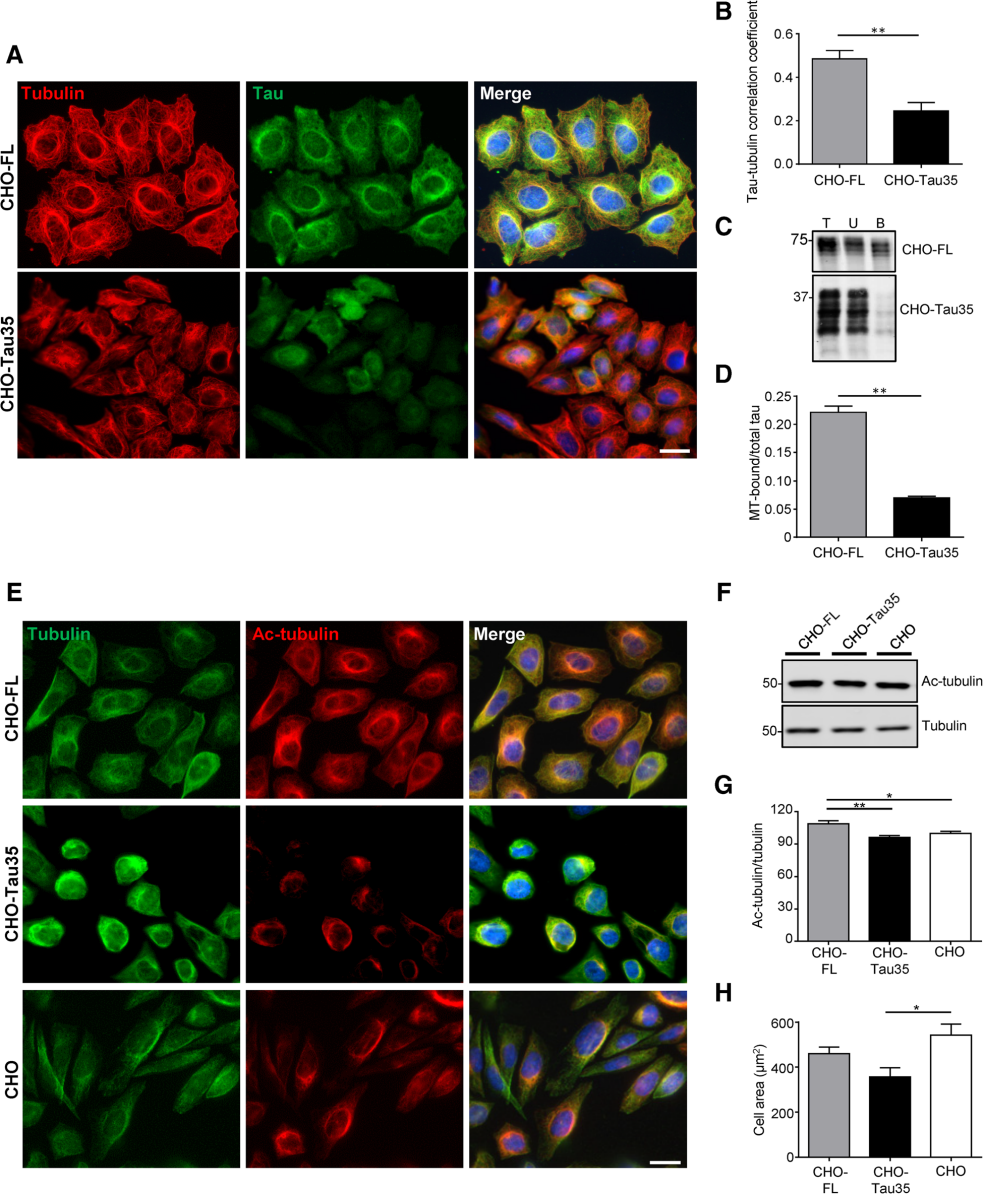
Reduced microtubule organization in CHO-Tau35 cells. **a** Immunofluorescence of methanol-fixed CHO-FL and CHO-Tau35 cells, labeled with antibodies to α-tubulin (red), tau (green) and Hoescht 33,342 (blue, nuclei). Scale bar = 20 μm. **b** Graph showing the correlation (Pearson’s) of tau colocalization with microtubules in CHO-FL and CHO-Tau35 cells. Values represent mean ± S.E.M, *n* = 80 cells from 3 independent experiments, Student’s t-test, **P < 0.01. **c** Western blots of total cell lysate (T), unbound (U), and microtubule-bound (B) fractions of CHO-FL and CHO-Tau35 cells probed with antibody to total tau. Molecular weight markers (kDa) are shown on the left. **d** Graph showing the ratio of tau in the microtubule (MT)-bound fraction relative to total tau in CHO-FL and CHO-Tau35 cell lysates. Values represent mean ± S.E.M., *n* = 3, Student’s t-test, **P < 0.01. **e** Immunofluorescence of paraformaldehyde-fixed CHO-FL, CHO-Tau35, and untransfected CHO cells labelled with antibodies to α-tubulin (green), acetylated α-tubulin (red) and Hoechst 33342 (blue). Scale bar = 20 μm. **f** Western blots of CHO-FL, CHO-Tau35 and CHO cell lysates probed with antibodies recognizing acetylated and total α-tubulin. Molecular weight markers (kDa) are shown on the left. **g** Graph showing the ratio of acetylated/total α-tubulin in CHO-FL and CHO-Tau35 cells, relative to CHO cells (100%). Values represent mean ± S.E.M., *n* = 4, one-way ANOVA, *P < 0.05, **P < 0.01. **h** Graph showing the areas of CHO-FL, CHO-Tau35 and CHO cells, mean ± S.E.M. A minimum of 50 cells were measured for each cell line. One-way ANOVA, *P < 0.05

Since increased tau phosphorylation reduces microtubule binding, we investigated the ability of Tau35 to stabilize microtubules. CHO-FL and CHO-Tau35 cells were fixed with methanol to remove soluble cytosolic proteins and visualize the cytoskeleton by immunocytochemistry. Expression of FL-tau induced formation of microtubule bundles and robust co-localization of tau with microtubules, neither of which were apparent in CHO-Tau35 cells (Fig. 2a). Indeed, Tau35 immunoreactivity markedly decreased after methanol fixation compared to that in paraformaldehyde-fixed cells, which preserves cytosolic proteins (Fig. 2a and Additional file 2: Figure S1). Colocalization analyses of the FL-tau/α-tubulin and Tau35/α-tubulin immunofluorescence in the two cell lines demonstrated a significantly reduced correlation of Tau35 with microtubules in CHO cells (Fig. 2b, *P* < 0.01). Thus, unlike FL-tau, Tau35 localizes predominantly in the cytosol, rather than binding to and stabilizing microtubules. The reduced ability of Tau35 to bind to microtubules was confirmed using an in situ microtubule binding assay. FL-tau was present in both microtubule-bound and unbound fractions, with approximately 22% of total tau bound to microtubules (Fig. 2 c, d). In contrast, approximately 6% of Tau35 was present in the microtubule-bound fraction in CHO-Tau35 cells. These results demonstrate a significant reduction in the ability of Tau35 to bind to microtubules, despite the presence of an intact microtubule-binding domain.

To determine the effect of Tau35 on microtubule stability, CHO-FL, CHO-Tau35 and CHO cells were fixed with paraformaldehyde and labelled with antibodies to total and acetylated (Lys40) α-tubulin (Fig. 2e). Only a minor proportion of the microtubule population was acetylated in CHO-Tau35 and CHO cells (Fig. 2e), whereas expression of FL-tau resulted in the formation of an ordered array of microtubules in CHO cells, and acetylated α-tubulin largely overlapped with microtubules detected by α-tubulin antibody (Fig. 2e). These findings indicate that tubulin acetylation is elevated in CHO-FL cells, likely due to increased microtubule stabilization. Acetylated α-tubulin in CHO-Tau35 cells was located mainly in perinuclear regions, coincident with the more intense localization of α-tubulin, and with a similar distribution to CHO cells (Fig. 2e). Analysis on western blots revealed increased acetylated α-tubulin in CHO-FL cells compared to CHO-Tau35 and CHO cells (Fig. 2f, g). Additionally, CHO-Tau35 cells exhibited a rounder morphology than the other two cell types (Fig. 2e), indicating a reduction in the organization and extent of the microtubule cytoskeleton. Disruption of microtubules in CHO-Tau35 cells was further evidenced by the finding that the area of CHO-Tau35 cells was significantly less than CHO cells (Fig. 2h). Taken together, these results show that, despite containing all four microtubule-binding repeats, Tau35 has a reduced ability to bind to and stabilize microtubules.

### Microtubule organization in CHO-Tau35 cells is not restored by increasing tubulin acetylation or decreasing tau phosphorylation

**Fig. 3:**
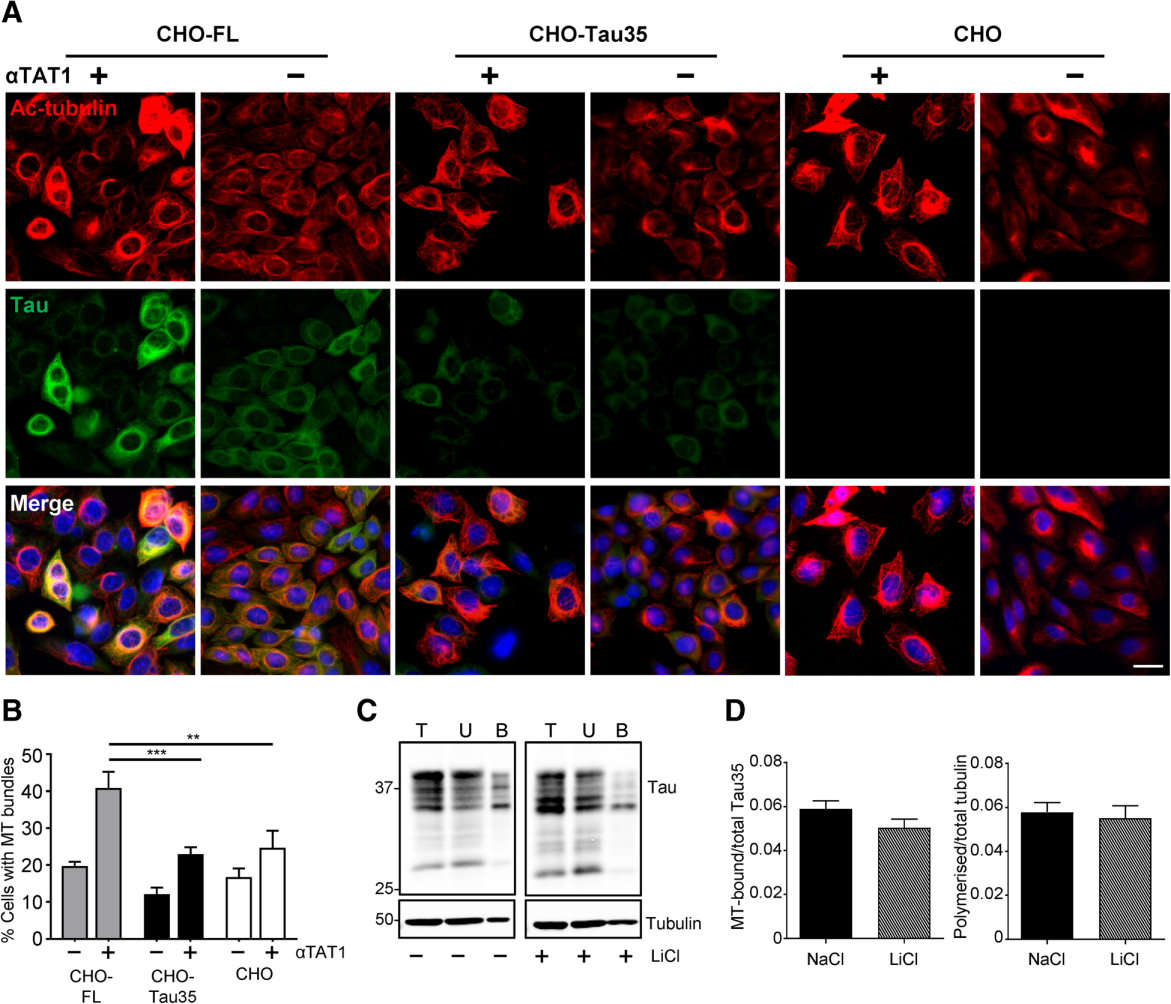
Microtubule organization in CHO-Tau35 cells is not restored by increasing tubulin acetylation or decreasing tau phosphorylation. **a** CHO-FL, CHO-Tau35 and CHO cells were transiently transfected with a plasmid expressing α-tubulin N-acetyltransferase 1 (αTAT1, **+**) or without plasmid (**−**). Panels show immunofluorescence of methanol-fixed cells labelled with antibodies to acetylated α-tubulin (red), tau (green), and Hoechst 33342 (blue, nuclei). Scale bar = 20 μm. **b** Graph showing the percentage of cells exhibiting microtubule (MT) bundles in CHO-FL, CHO-Tau35 and CHO cells in the presence (**+**) or absence (**−**) of αTAT1. Values represent mean ± S.E.M. A minimum of 150 cells were counted for each transfection condition; two-way ANOVA, **P < 0.01; ***P < 0.001. **c** Western blots of tau and α-tubulin in total cell lysate (T), unbound (U) and MT-bound (B) fractions of CHO-Tau35 cells treated with 5 mM LiCl (**+**) or 5 mM NaCl (**−**) for 24 h and probed with antibodies to tau or α-tubulin. **d** Graphs show the amount of tau present in the MT-bound fractions, relative to total Tau35 (left), and the amount of polymerized α-tubulin relative to total α-tubulin (right) in CHO-Tau35 cells, following exposure to NaCl (control) or LiCl. Values represent mean ± S.E.M., *n* = 3. Student’s t-test, *P < 0.05, **P < 0.01

To determine whether enhancing α-tubulin acetylation could reverse the Tau35-induced loss of microtubule stabilization, cells were transiently transfected with a plasmid expressing α-tubulin N-acetyltransferase 1 (αTAT1) to promote tubulin acetylation [5, 17, 37]. Expression of αTAT1 was confirmed on western blots 48 h after transfection (see Additional file 2: Figure S2). Methanol-fixed cells labelled with antibodies to acetylated α-tubulin and tau showed that exogenous expression of αTAT1 increased α-tubulin acetylation in all three cell lines (Fig. 3a). Notably, αTAT1 significantly enhanced the formation of highly acetylated microtubule bundles that formed perinuclear rings in CHO-FL cells (Fig. 3a, b). FL-tau co-localized with microtubule rings following methanol fixation (Fig. 3a). In contrast, increased α-tubulin acetylation resulted in little microtubule bundling and the extent of co-localization of acetylated α-tubulin with tau was also limited in CHO-Tau35 cells (Fig. 3a), demonstrating that the reduced microtubule-binding ability of Tau35 in not restored by increasing tubulin acetylation. Similar to CHO-Tau35 cells, increasing α-tubulin acetylation in CHO cells also resulted in only limited microtubule bundling in the absence of tau (Fig. 3a). These results indicate that in contrast to FL-tau, Tau35 lacks the ability to promote microtubule bundling in cells.

Phosphorylation of tau by kinases including GSK3, negatively affects interactions between tau and microtubules [10, 15, 46]. To investigate whether the decreased microtubule binding ability of Tau35 is due to its increased phosphorylation state (Fig. 1), CHO-Tau35 cells were treated with 5 mM LiCl for 24 h to inhibit GSK3-mediated tau phosphorylation. Western blots of CHO-Tau35 cell lysates probed with PHF1 antibody confirmed that LiCl reduced Tau35 phosphorylation by approximately 75% at residues Ser396/Ser404 and caused a shift of tau species to lower apparent molecular weight, confirming the highly phosphorylated nature of Tau35 (see Additional file: Figure S3). Despite the clear reduction in Tau35 phosphorylation, LiCl did not affect the proportion of either microtubule-bound Tau35, or polymerized α-tubulin in CHO-Tau35 cells (Fig. 3c, d). Therefore, decreasing the phosphorylation state of Tau35 appears to be insufficient to restore its interaction with microtubules. Hence, increased phosphorylation of Tau35 appears unlikely to be a major factor involved in its inability to bind to microtubules in cells.

### Divergent effects of FL-tau and Tau35 on insulin signaling via Akt/GSK3

**Fig. 4:**
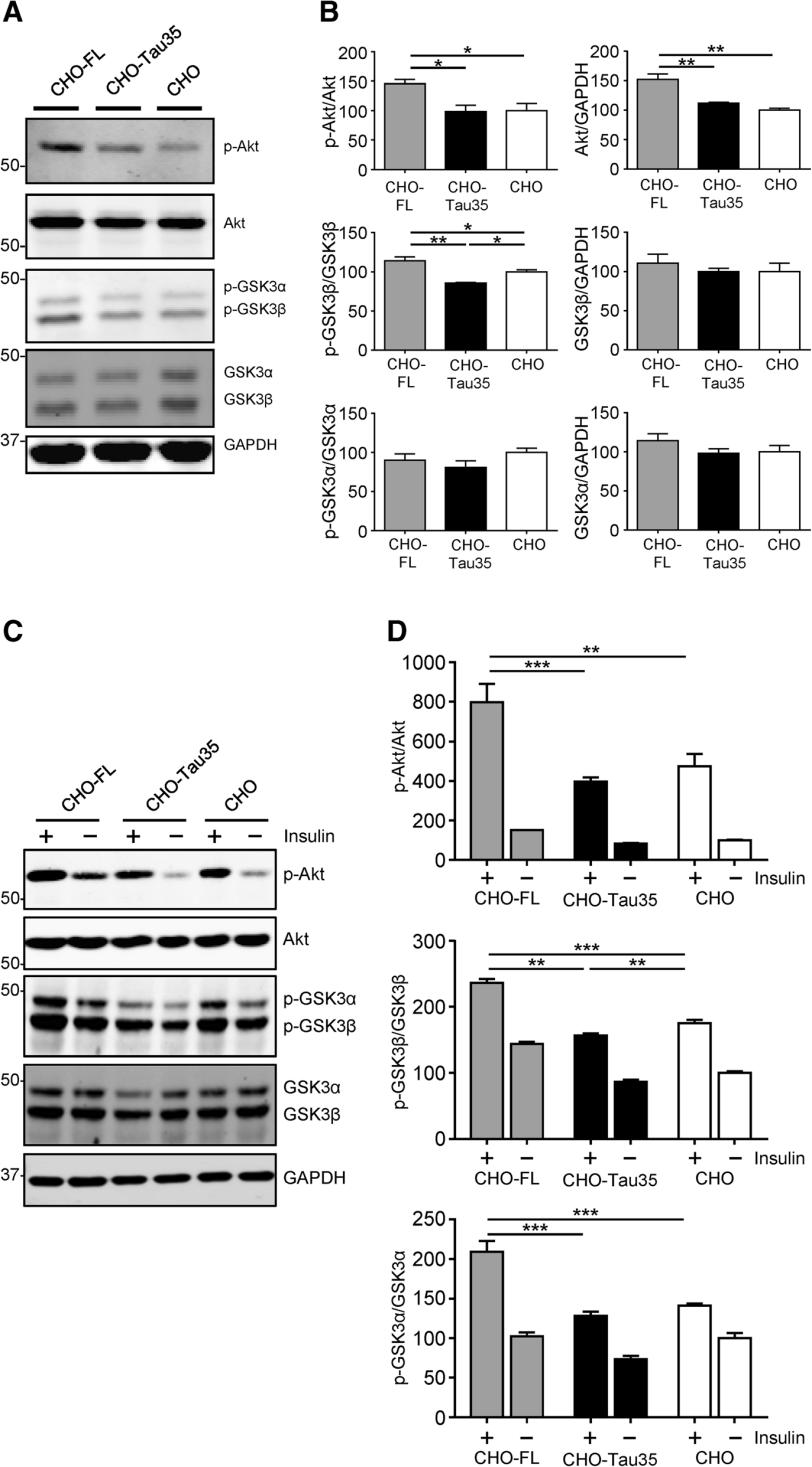
Differing effects of FL-tau and Tau35 on Akt/GSK3 signaling. **a** Western blots of CHO-FL, CHO-Tau35 and untransfected CHO cell lysates probed with antibodies against phosphorylated and total Akt, phosphorylated and total GSK3α/β, and glyceraldehyde 3-phosphate dehydrogenase (GAPDH). Molecular weight markers (kDa) are shown on the left. **b** Graphs show the percentage of phosphorylated/total Akt and phosphorylated/total GSK3α/β, relative to untransfected CHO cells (100%). Values represent mean ± S.E.M., *n* = 4, one-way ANOVA, *P < 0.05, **P < 0.01. **c** Western blots of lysates of CHO-FL, CHO-Tau35 and untransfected CHO cell lysates treated with (**+**) or without (**−**) 100 nM insulin for 30 min. Blots were probed with antibodies against phosphorylated and total Akt, phosphorylated and total GSK3α/β, and GAPDH. Molecular weight markers (kDa) are shown on the left. **d** Graphs show phosphorylated/total Akt, phosphorylated/total GSK3β, and phosphorylated/total GSK3α. Data are displayed as percentage change compared to the untreated CHO cells (100%). Values represent mean ± S.E.M., *n* = 6, two-way ANOVA, **P < 0.01, ***P < 0.001

The increased phosphorylation of Tau35 in cells suggests altered activity of tau kinases. Therefore, we determined the activity of the candidate tau kinase GSK3, and its upstream inhibitor Akt (protein kinase B), in CHO-FL, CHO-Tau35 and CHO cells. CHO-FL cells exhibited enhanced total and phosphorylated Akt, indicative of increased activity, in comparison to CHO-Tau35 and CHO cells (Fig. 4a, b). Correspondingly, inhibitory phosphorylation of GSK3β at Ser9 was augmented in CHO-FL cells compared to CHO-Tau35 and CHO cells (Fig. 4b). We also detected reduced inhibitory phosphorylation of GSK3β, indicating activation of GSK3β in the presence of Tau35 (Fig. 4a, b). The total amount and phosphorylation state of GSK3α was comparable in all three cell lines (Fig. 4a, b). These data indicate that FL-tau and Tau35 exert differing effects on the Akt/GSK3 pathway, with FL-tau promoting the steady-state amount of Akt and also increasing its activation state, thereby inactivating GSK3β. In contrast, Tau35 lacks the capacity to activate Akt and reduce the activity of GSK3β, suggesting a potential mechanism for the increased phosphorylation of Tau35.

Since the Akt/GSK3 pathway is a critical mediator of the insulin signaling pathway, we investigated the effects of FL-tau and Tau35 on insulin responsiveness in CHO cells. Lysates from CHO-FL, CHO-Tau35 and CHO cells treated with 100 nM insulin for 30 min were analyzed on western blots (Fig. 4c). Insulin significantly increased Akt phosphorylation in all cell lines (Fig. 4c, d). CHO-FL cells exhibited the greatest (4.4-fold) Akt activation, whereas in both CHO-Tau35 cells and CHO cells, insulin resulted in approximately 3.7-fold activation of Akt. In line with Akt activation, insulin promoted inhibitory phosphorylation of GSK3α and GSK3β in cell lines (Fig. 4d). As previously observed with Akt activation, insulin-mediated inhibitory phosphorylation of GSK3α and GSK3β was most affected in CHO-FL cells. In contrast, inhibitory phosphorylation of GSK3β in response to insulin was significantly lower in CHO-Tau35 cells than in CHO-FL and CHO cells. Insulin-mediated GSK3α activation was similar in both CHO-Tau35 and CHO cells, whereas it was significantly elevated in CHO-FL cells. These findings suggest that, compared to CHO cells expressing FL-tau, Tau35 impairs insulin responsiveness, repressing both Akt activation and inhibitory phosphorylation of GSK3, particularly GSK3β.

### Tau35 impairs insulin signaling through activation of mTORC1/S6K1 signaling

**Fig. 5:**
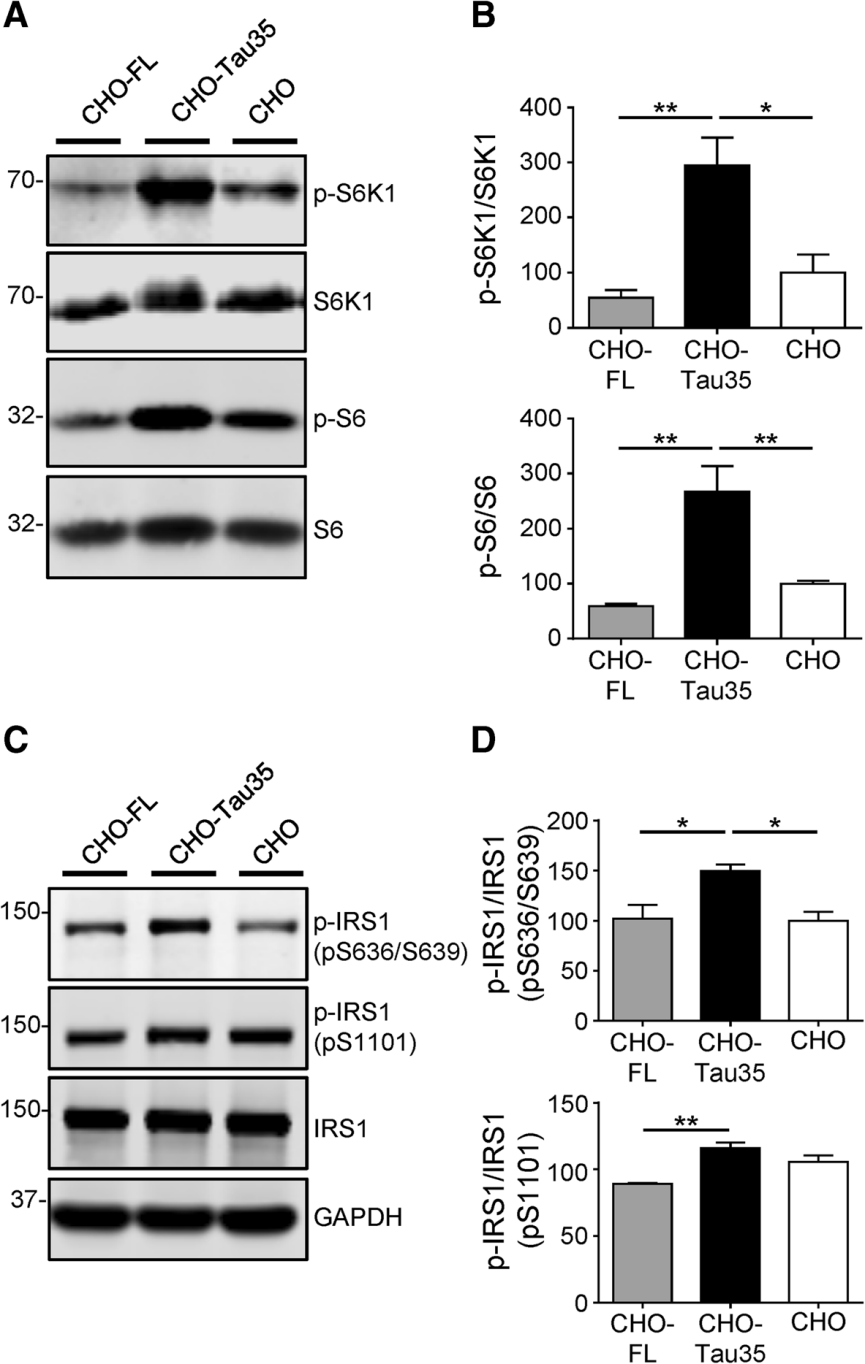
mTORC1/S6K1-mediated IRS1 inhibition by Tau35. **a** Western blot analysis of downstream effectors of mTORC1. CHO-FL, CHO-Tau35 and untransfected CHO cell lysates probed with antibodies to phosphorylated/total ribosomal protein S6 kinase beta-1 (S6K1) and phosphorylated/total S6 ribosomal protein (S6). Molecular weight markers (kDa) are shown on the left. **b** Graphs show the relative amounts of phosphorylated/total S6K1 and phosphorylated/total S6. Data are displayed as percentage change compared to untransfected CHO cells (100%). **c** Western blot analysis of IRS1 activity. CHO-FL, CHO-Tau35 and untransfected CHO cell lysates probed with antibodies to phosphorylated/total insulin receptor substrate 1 (IRS1), and GAPDH. Molecular weight markers (kDa) are shown on the left. **d** Graphs show the relative amounts of phosphorylated (Ser636/Ser639)/total IRS1, and phosphorylated (Ser1101)/total IRS1. Data are displayed as percentage changes compared to untransfected CHO cells (100%). Values represent mean ± S.E.M., *n* = 4–6, one-way ANOVA, *P < 0.05, **P < 0.01

Repression of insulin signaling is associated with activation of mTORC1 and its downstream effector ribosomal protein S6 kinase β-1 (S6K1), which is phosphorylated and activated by mTORC1-mediated phosphorylation at Thr389. mTORC1/S6K1 signaling negatively regulates insulin signaling through inhibitory phosphorylation of insulin receptor substrate 1 (IRS1) [35]. When assessed on western blots, S6K1 phosphorylation in CHO-Tau35 cells was approximately 3-fold higher than that in CHO cells, whereas it was unchanged in CHO-FL cells (Fig. 5a, b), indicating a selective upregulation of S6K1 activity by Tau35. Phosphorylation of the S6K1 substrate, S6 ribosomal protein, also increased approximately 3-fold in CHO-Tau35 cells and remained unchanged in CHO-FL cells (Fig. 5a, b). These data suggest that mTORC1/S6K1 signaling is significantly upregulated in CHO-Tau35 cells, and this appears to be a gain of function of Tau35 that differentiates it from FL-tau.

Amongst the inhibitory target phosphorylation sites on human IRS1, Ser636/Ser639 and Ser1101 (equivalent to rodent Ser632/Ser635 and Ser1097) are phosphorylated following mTORC1/S6K1 activation [50, 51]. Enhanced activation of S6K1 in CHO-Tau35 cells promoted Ser636/Ser639 phosphorylation of IRS1, compared to both CHO and CHO-FL cells (Fig. 5c, d). S6K1 also increased IRS1 Ser1101 phosphorylation in CHO-Tau35 cells compared to CHO-FL cells (Fig. 5c, d). These results indicate that the disrupted insulin signaling apparent in CHO-Tau35 cells may be caused by mTORC1/S6K1-mediated inhibitory phosphorylation of IRS1, which does not occur with FL-tau.

### Tau35 induces the unfolded protein response

**Fig. 6:**
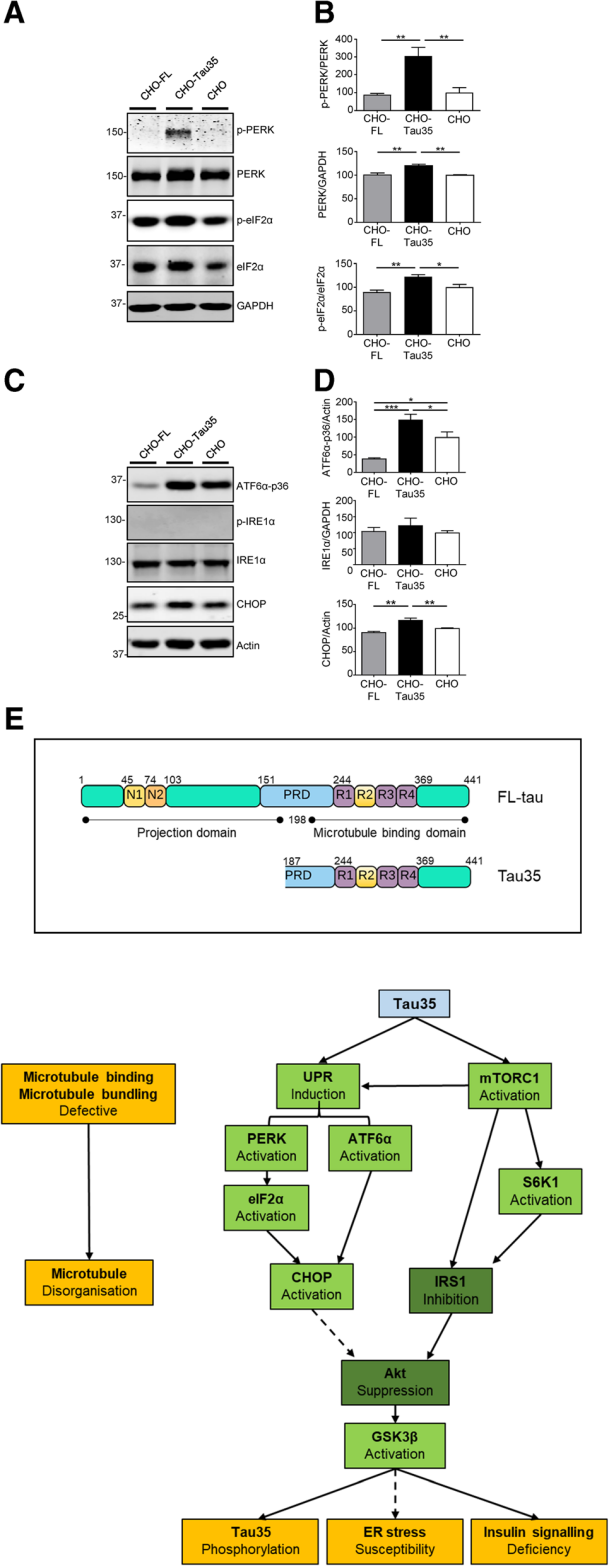
Induction of the unfolded protein response in CHO-Tau35 cells. **a** Western blot analysis of components of the PERK branch of the UPR. CHO-FL, CHO-Tau35 and CHO cell lysates were probed with antibodies to phosphorylated/total PERK, phosphorylated/total eIF2α, and GAPDH. Molecular weight markers (kDa) are shown on the left. **b** Graphs show the relative amounts of phosphorylated/total PERK, total PERK/GAPDH, and phosphorylated/total eIF2α. Data are displayed as percentage changes compared to untransfected CHO cells (100%). Values represent mean ± S.E.M., *n* = 4–6, one-way ANOVA, *P < 0.05, **P < 0.01. **c** Western blots of ATF6, IRE1 and CHOP components of the UPR. CHO-FL, CHO-Tau35 and CHO cell lysates were probed with antibodies to cleaved ATF6α (36 kDa), phosphorylated/total IRE1α, CHOP and β-actin. **d** Graphs show the relative amounts of cleaved ATF6α (36 kDa)/β-actin, total IRE1α/GAPDH, and total CHOP/β-actin. Data are displayed as percentage changes compared to untransfected CHO cells (100%). Values represent mean ± S.E.M., *n* = 4–6, one-way ANOVA, *P < 0.05, **P < 0.01, ***P < 0.001. **e** The major domains and amino acid numbering of human FL-tau and Tau35 are illustrated (upper panel) showing the projection (amino acids 1–198) and microtubule binding (amino acids 199–441) domains of FL-tau. N1 and N2 correspond to the alternatively spliced amino acid sequences encoded by exons 2 and 3, respectively. The proline-rich domain (PRD, amino acids 151–243) is followed by the microtubule binding repeat region (R1-R4, amino acids 244–368), including the alternatively spliced sequence encoded by exon 10 (R2). The scheme in the lower panel indicates potential mechanisms by which Tau35 may be involved in the development of tauopathy. Tau35 has a reduced ability to bind to and stabilize microtubules, which compromises microtubule organization (loss of function). Potential gains of toxic function by Tau35 include triggering the activation of multiple branches of the unfolded protein response (UPR) and an increase in CHOP, which may lead to the downstream suppression of Akt activity. The accumulation of unfolded Tau35 results in activation of GSK3β through perturbation of Akt signaling and increased tau phosphorylation. In parallel, Tau35 expression activates mTORC1-S6K1 signaling, resulting in inhibitory phosphorylation of IRS1 and suppression of Akt, rendering CHO-Tau35 cells less responsive to insulin

Since mTORC1 activation can induce the UPR, we investigated the status of the UPR in CHO cells expressing FL-tau and Tau35. Activation of PRKR-like endoplasmic reticulum kinase (PERK) is observed in CHO-Tau35 cells, as demonstrated by increases in both total and phosphorylated PERK (Fig. 6a, b). In marked contrast, PERK activation was negligible in CHO-FL and CHO cells (Fig. 6a, b). PERK activation leads to phosphorylation of eukaryotic translation initiation factor 2α (eIF2α), which was also increased in CHO-Tau35 cells (Fig. 6a, b). These results indicate that Tau35, but not FL-tau, selectively activates the PERK branch of the UPR.

In parallel to PERK activation, we also found an enrichment of the 36 kDa fragment of activating transcription factor 6α (ATF6α-p36) in CHO-Tau35 cells (Fig. 6c, d), indicating activation of the ATF6α branch of the UPR [23, 28, 36]. Notably, cleavage of ATF6α was significantly reduced in CHO-FL cells, indicating potential suppression of this branch of the UPR by FL-tau. We were unable to detect phosphorylation (activation) of inositol-requiring enzyme 1α (IRE1α) in any of the three cell lines and there were no differences in the total amount of IRE1α present (Fig. 6c, d), suggesting that this branch of the UPR is not activated by either Tau35 or FL-tau.

CCAAT-enhancer-binding protein homologous protein (CHOP) is an integrated transcriptional target that lies downstream of both the PERK and ATF6 branches of the UPR. Western blots of CHOP showed a corresponding increase in CHO-Tau35 cells, whereas the amount of CHOP in CHO-FL cells was unchanged (Fig. 6c, d). Furthermore, induction of endoplasmic reticulum (ER) stress with thapsigargin, resulted in sensitized activation of both PERK and IRE1α in CHO-Tau35 cells compared with both CHO-FL and CHO cells (see Additional file 2: Figure S4). These results suggest that CHO-Tau35 cells are more sensitive to ER stress, which is widely seen in cells with chronic mTORC1 activation [13, 24]. Taken together, these results support the view that expression of Tau35 but not FL-tau, selectively activates the PERK and ATF6α branches of the UPR, without affecting IRE1α signaling.

## Discussion

We previously identified a 35 kDa C-terminal tau fragment termed Tau35 (residues 187–441 of FL-tau), in 4R human tauopathy brain [53]. Tau35 is generated by cleavage of human tau, resulting in a tau fragment that lacks the N-terminal domain and part of the proline-rich domain, but contains all four microtubule-binding repeats and an intact C-terminus (Fig. 6e). Minimal expression of Tau35 in transgenic mice causes several key features of human tauopathy [7]. Here we investigated the molecular mechanisms underlying the development of disease-related phenotypes using cells stably expressing Tau35.

Tau phosphorylation plays a key role in regulating tau localization and function, and aberrant phosphorylation of tau reduces its ability to bind to microtubules [16, 46]. When expressed in CHO cells, Tau35 displayed elevated phosphorylation at several epitopes associated with the development of human tauopathy, in which aggregates of highly phosphorylated and fragmented tau are present [4, 53], highlighting the relevance of this Tau35 cell model to human tauopathy.

Tau35 has a reduced ability to bind to microtubules, despite the presence of all four microtubule binding repeats (residues 244–401 of human FL-tau) and an intact C-terminus. Moreover, reducing Tau35 phosphorylation using lithium did not increase the interaction between Tau35 and microtubules, suggesting that the reduced binding was not due to increased phosphorylation of Tau35 in CHO cells. Our findings therefore support the view that the reduced microtubule binding ability of Tau35 is due to the absence of amino acid sequences present in the N-terminal half of tau. These results parallel those of others showing that N-terminally cleaved tau species display altered interactions with microtubules [11, 31, 57] and indicate that the N-terminal region of tau is important for its association with microtubules. It is possible that an extended region of tau, encompassing domains outside the microtubule binding and flanking regions, may be required in order to facilitate microtubule binding and stabilization since truncated forms of tau corresponding to residues 1–255 and 256–441 also exhibit reduced abilities to polymerize microtubules [57]. Notably, unlike Tau35, a different tau fragment comprising residues 124–441, displays an increased ability to bind and stabilize microtubules compared to FL-tau [11]. Taken together, these findings indicate that a sequence of amino acids located between residues 124–186 of FL-tau may be critical for its interaction with microtubules.

Tau35 was unable to induce significant microtubule bundling, even after enhancing tubulin acetylation, consistent with the proposal that microtubule bundling is promoted by complementary intermolecular dimerization between the N-terminus and the proline-rich domains of tau [42]. Such a model provides an explanation for this loss of function of Tau35, since it lacks N-terminal tau residues, which may be required for regulating microtubule organization.

The adverse effects of Tau35 on insulin signaling also distinguish it from FL-tau. In particular, FL-tau interacts with and reduces the activity of phosphatase and tensin homologue on chromosome 10 (PTEN), thereby promoting Akt activation [30]. Notably, knocking out tau also reduces Akt activity and impairs the hippocampal response to insulin [30]. Our finding of increased Akt activity in the presence of FL-tau supports this view, whereas cleavage of tau to generate Tau35 could potentially prevent or perturb its interaction with PTEN, attenuating Akt activation and resulting in enhanced GSK3 activity. Defective inactivation of GSK3β in CHO-Tau35 cells was also observed in CHO-Tau35 cells exposed to insulin. Whereas FL-tau facilitates insulin signaling, attenuated Akt phosphorylation in response to insulin in CHO-Tau35 cells suggests the induction of insulin resistance.

We also identified activation of mTORC1/SK61 signaling as a potential mediator of the adverse impact of Tau35 on insulin signaling in CHO-Tau35 cells, resulting in IRS1 phosphorylation. Altered IRS1 phosphorylation and insulin resistance have been reported in several tauopathies, including Alzheimer’s disease, progressive supranuclear palsy and corticobasal degeneration [54]. However, the mechanisms leading from pathological changes in tau, to insulin resistance in human tauopathy are not well understood. Notably, upregulation of mTORC1 activity increases both tau phosphorylation and tau pathology [9]. Our data therefore support the view that Tau35 may trigger inhibitory phosphorylation of IRS1 through activation of mTORC1/S6K1 signaling, which in turn exacerbates phosphorylation of Tau35.

Intriguingly, we identified chronic activation of the PERK and ATF6 branches of the UPR induced by Tau35, which has also been found in human tauopathy [8, 39, 47]. Activation of the PERK and ATF6α branches of the UPR lead to expression of pro-apoptotic factor CHOP, which is also elevated by Tau35 [21, 25]. Interestingly, the IRE1α branch of the UPR does not appear to be affected, indicating selectivity in Tau35-induced UPR activation. Prolonged PERK signaling impairs cell proliferation and promotes apoptosis, whereas IRE1α signaling enhances cell proliferation [27]. Such divergence in the activation of PERK and IRE1α may be indicative of persistent ER stress [26, 27] in CHO-Tau35 cells, which ultimately results in an imbalance between detrimental and protective effects of UPR activation. It has been suggested that PERK can facilitate the translation and activation of ATF6α [48], thus, it will be of interest to examine whether Tau35 directly triggers ATF6α activation, or whether this is the result of prior PERK activation. Tau35 also renders CHO cells more susceptible to thapsigargin-mediated activation of the UPR, which has been linked to accumulation of abnormally phosphorylated tau [1, 29]. The mechanisms that contribute to UPR activation in tauopathy are unclear. It has been proposed that soluble tau oligomers are the driving force behind tau-induced ER stress [1] and these could impair ER-associated degradation, resulting in UPR activation [14, 38].

Activation of both mTORC1/S6K1 and UPR signaling are associated with neurodegenerative disease [3] and importantly, crosstalk between these two pathways is increasingly recognised. Such interactions include ATF6α-mediated upregulation of mTORC1 [43] and UPR activation contributing to insulin resistance [6, 33]. Given the inter-dependence of these two pathways, suppressed insulin signaling may be the synergistic consequence of activation of both the UPR and mTORC1/S6K1 pathways in CHO-Tau35 cells and potentially also in human tauopathy brain.

## Conclusions

In summary, we propose a mechanism in which N-terminal cleavage of tau leads to the development and progression of tau pathology, compromising the ability of tau to facilitate microtubule organization, disrupting microtubule integrity, microtubule assembly, and binding to microtubules (Fig. 6e). Moreover, Tau35 triggers activation of the mTORC1-S6K1 and UPR pathways, undermining insulin signaling through inhibitory phosphorylation of IRS1. Moreover, our findings highlight the importance of the N-terminal half of tau for its physiological function since lack of this region disrupts insulin-Akt-GSK3β signaling, fuels tau phosphorylation and increases susceptibility to ER stress. Consequently, accumulation of potentially pathogenic tau fragments such as Tau35, results in a perpetuating cycle of tau pathology that disrupts cellular function and leads to the demise of neurons in the tauopathies. Thus, cleavage of tau may be a key event linking tau pathology to disruptions in insulin sensitivity and aberrant cell signaling. Our findings provide several potential targets for therapeutic intervention in the tauopathies and demonstrate the utility of the CHO-Tau35 cell model as a tool for drug discovery.

## Additional files

Additional file 1: **Table S1.** Antibodies used for western blots and immunohistochemistry. (PDF 79 kb)

Additional file 2: **Figure S1.** Expression of tau in paraformaldehyde-fixed CHO-FL and CHO-Tau35 cells. Immunofluorescence of paraformaldehyde-fixed CHO-FL, CHO-Tau35, and untransfected CHO cells labeled with antibodies to α-tubulin (red), total tau (green), and Hoechst 33342 (blue). Scale bar = 20 μm. **Figure S2.** Validation of exogenous expression of α-tubulin N-acetyltransferase 1 (αTAT1). Western blots of CHO-FL, CHO-Tau35 and CHO cell lysates transfected with (+) or without (−) a plasmid encoding αTAT1. Blots were probed with antibodies recognizing αTAT1, acetylated and total α-tubulin. Molecular weight markers (kDa) are shown on the left. **Figure S3.** LiCl treatment reduces phosphorylation of Tau35. Western blots of CHO-Tau35 cell lysates treated with 5 mM NaCl (−) or 5 mM LiCl (+) for 24 h and probed with antibodies against total and phosphorylated (PHF1) tau. Graphs show the ratio of phosphorylated/total tau in the presence of NaCl (control) or LiCl. Values represent mean ± S.E.M., *n* = 3. Student’s t-test, *P < 0.05. **Figure S4.** Thapsigargin-induced UPR activation in CHO-FL, CHO-Tau35 and CHO cells. **a** Western blots of CHO-FL, CHO-Tau35 and CHO cell lysates treated with (+) or without (−) 800 nM thapsigargin (TG) for 5 h. Blots were probed with antibodies recognizing phosphorylated/total PERK, phosphorylated/total IRE1α, and GAPDH. Molecular weight markers (kDa) are shown on the left. **b** Graphs show the relative amounts of phosphorylated/total PERK, and phosphorylated/total IRE1α after thapsigargin treatment. Data are displayed as percentage change compared to TG-treated CHO cells (100%). Values represent mean ± S.E.M., *n* = 4, two-way ANOVA, *P < 0.05, **P < 0.01. (PDF 332 kb)

## Abbreviations

Akt: Protein kinase B
ANOVA: Analysis of variance
ATF6α: Activating transcription factor 6α
ATF6α-p36: ATF6α-36 kDa fragment
CHO: Chinese hamster ovary
CHOP: CCAAT-enhancer-binding protein homologous protein
eIF2α: Eukaryotic initiation factor 2α
ER: Endoplasmic reticulum
FL: Full-length 2N4R human tau
GAPDH: Glyceraldehyde 3-phosphate dehydrogenase
GSK3α/β: Glycogen synthase kinase-3α/β
IgG: Immunoglobulin G
IRE1α: Inositol-requiring enzyme 1α
IRS1: Insulin receptor substrate 1
Lys: Lysine
mTORC1: Nutrient sensor mammalian target of rapamycin complex 1
PBS: Phosphate-buffered saline
PERK: PRKR-like endoplasmic reticulum kinase
PTEN: Phosphatase and tensin homologue on chromosome 10
S6K1: Ribosomal protein S6 kinase β-1
Ser: Serine
TBST: Tris-buffered saline/0.2% (*v*/v) Tween 20
Thr: Threonine
UPR: Unfolded protein response
αTAT1: α-Tubulin N-acetyltransferase 1
