# Supplementary material for: A pathogenic tau fragment compromises microtubules, disrupts insulin signaling and induces the unfolded protein response
Source: Acta Neuropathol Commun. 2019 Jan 3;7:2. doi: 10.1186/s40478-018-0651-9 (PMC6318896; doi:10.1186/s40478-018-0651-9)
Supplement: Supplementary file 1 — Table S1. Antibodies used for western blots and immunohistochemistry. (PDF 79 kb) [file 40478_2018_651_MOESM1_ESM.pdf]

Additional file 1: Table S1. Antibodies used for western blots and immunohistochemistry

Primary Antibodies				
**Antibody**	**Antigen/Epitope**	**WB**	**ICC**	**Source**
K9JA	C-terminal half of tau	1/10,000	1/1,000	DAKO
PHF1	Tau phosphorylated at S396/S404	1/5,000	-	Professor P. Davies [1]
AT8	Tau phosphorylated at S202/T205	1/3,000	-	Thermo Fisher Scientific
AT180	Tau phosphorylated at T231	1/500	-	Innogenetics
AC15	β-Actin	1/10,000	-	Abcam
Actin	β-Actin (Ab8227)	1/10,000	-	Abcam
DM1A	α-Tubulin	1/1,000	1/1,000	Sigma
Acetylated tubulin	Tubulin acetylated at Lys40	1/2,000	1/200	Santa Cruz Biotechnology
α-Tubulin	α-Tubulin	1/1,000	1/200	Abcam
αTAT1	α-Tubulin N-acetyltransferase 1 (MEC-17)	1/500	-	Abcam
GSK3	Glycogen synthase kinase3α/β (GSK3α/β)	1/1,000	-	Santa Cruz Biotechnology
Phospho-GSK3	GSK3α/β phosphorylated at Ser21/Ser9	1/500	-	Cell Signaling Technology
Akt	Akt	1/1,000	-	Cell Signaling Technology
Phospho-Akt	Akt phosphorylated at Ser473	1/1,000	-	Cell Signaling Technology
PERK	PRKR-like endoplasmic reticulum kinase	1/1,000	-	Cell Signaling Technology
Phospho-PERK	PERK phosphorylated at Thr980	1/1,000	-	Cell Signaling Technology
eIF2α	Eukaryotic Initiation factor 2α	1/500	-	Cell Signaling Technology
Phospho-eIF2α	EIF2α phosphorylated at Ser51	1/500	-	Cell Signaling Technology
ATF6α	Amino acids 1-273 of human activating transcription factor 6	1/500	-	Novus Biologicals
IRE1α	Inositol-requiring enzyme 1α	1/1,000	-	Cell Signaling Technology
Phospho-IRE1α	IRE1α phosphorylated at S724	1/1,000	-	Novus Biologicals
CHOP	Amino acids 1-168 of full length CCAAT-enhancer-binding protein homologous protein	1/200	-	Santa Cruz Biotechnology
GAPDH	Glyceraldehyde 3-phosphate dehydrogenase	1/2,000	-	Santa Cruz Biotechnology
**Secondary Antibodies**				
**Antibody**	**Species Reactivity**	**WB**	**ICC**	**Source**
AlexaFluor 680	Anti-mouse IgG	1/10,000	-	Life Technologies
IRDye 800	Anti-rabbit IgG	1/10,000	-	Rockland Inc
AlexaFluor 488	Anti-rabbit IgG	-	1/500	Life Technologies
AlexaFluor 568	Anti-mouse IgG	-	1/500	Life Technologies

ICC = immunocytochemistry; IgG = immunoglobulin G; WB = western blot

Reference

1 Greenberg SG, Davies P, Schein JD, Binder LI (1992) Hydrofluoric acid-treated tau PHF proteins display the same biochemical properties as normal tau. J Biol Chem 267: 564-569
