# Supplementary material for: A pathogenic tau fragment compromises microtubules, disrupts insulin signaling and induces the unfolded protein response
Source: Acta Neuropathol Commun. 2019 Jan 3;7:2. doi: 10.1186/s40478-018-0651-9 (PMC6318896; doi:10.1186/s40478-018-0651-9)

Additional file 2: Figure S1

Expression of tau in paraformaldehyde-fixed CHO-FL and CHO-Tau35 cells

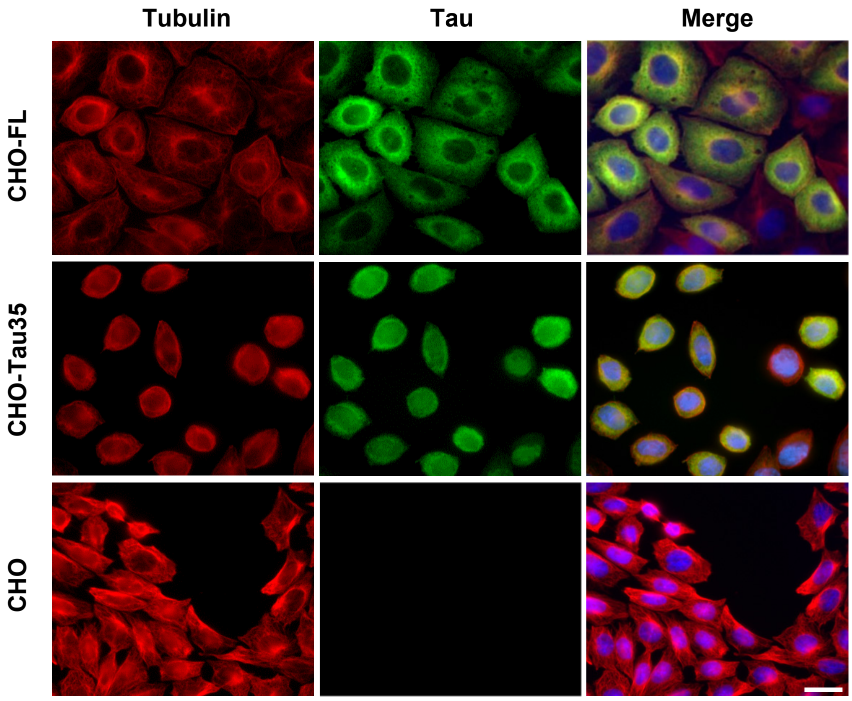

Additional file 2: Figure S2

Validation of exogenous expression of αTAT1

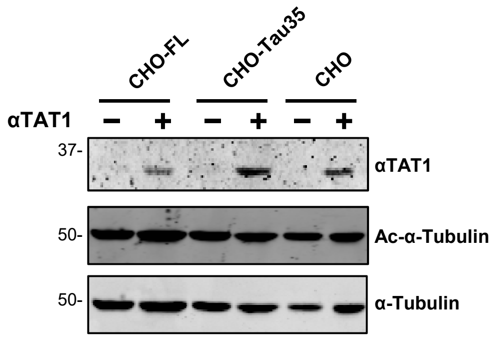

Additional file 2: Figure S3

LiCl treatment reduced phosphorylation of Tau35

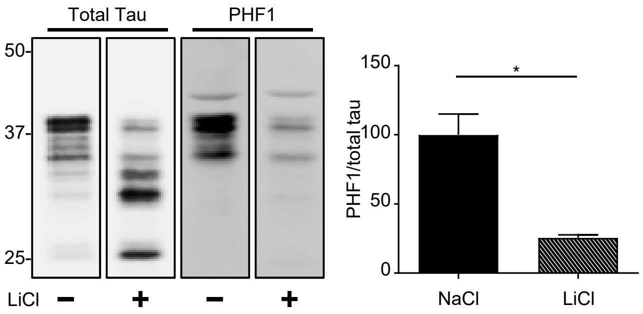

Additional file 2: Figure S4

Thapsigargin-induced UPR activation in CHO-FL, CHO-Tau35 and CHO cells

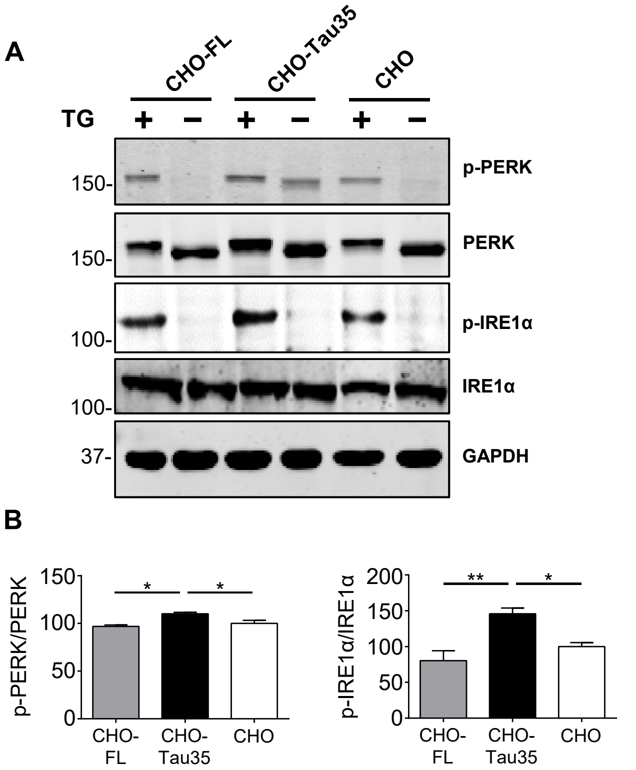

Supplement: Supplementary file 2 — Figure S1. Expression of tau in paraformaldehyde-fixed CHO-FL and CHO-Tau35 cells. Immunofluorescence of paraformaldehyde-fixed CHO-FL, CHO-Tau35, and untransfected CHO cells labeled with antibodies to α-tubulin (red), total tau (green), and Hoechst 33342 (blue). Scale bar = 20 μm. Figure S2. Validation of exogenous expression of α-tubulin N-acetyltransferase 1 (αTAT1). Western blots of CHO-FL, CHO-Tau35 and CHO cell lysates transfected with (+) or without (−) a plasmid encoding αTAT1. Blots were probed with antibodies recognizing αTAT1, acetylated and total α-tubulin. Molecular weight markers (kDa) are shown on the left. Figure S3. LiCl treatment reduces phosphorylation of Tau35. Western blots of CHO-Tau35 cell lysates treated with 5 mM NaCl (−) or 5 mM LiCl (+) for 24 h and probed with antibodies against total and phosphorylated (PHF1) tau. Graphs show the ratio of phosphorylated/total tau in the presence of NaCl (control) or LiCl. Values represent mean ± S.E.M., n = 3. Student’s t-test, *P < 0.05. Figure S4. Thapsigargin-induced UPR activation in CHO-FL, CHO-Tau35 and CHO cells. a Western blots of CHO-FL, CHO-Tau35 and CHO cell lysates treated with (+) or without (−) 800 nM thapsigargin (TG) for 5 h. Blots were probed with antibodies recognizing phosphorylated/total PERK, phosphorylated/total IRE1α, and GAPDH. Molecular weight markers (kDa) are shown on the left. b Graphs show the relative amounts of phosphorylated/total PERK, and phosphorylated/total IRE1α after thapsigargin treatment. Data are displayed as percentage change compared to TG-treated CHO cells (100%). Values represent mean ± S.E.M., n = 4, two-way ANOVA, *P < 0.05, **P < 0.01. (PDF 332 kb) [file 40478_2018_651_MOESM2_ESM.pdf]
